# The UBR-box and its relationship to binuclear RING-like treble clef zinc fingers

**DOI:** 10.1186/s13062-015-0066-5

**Published:** 2015-07-17

**Authors:** Gurmeet Kaur, Srikrishna Subramanian

**Affiliations:** CSIR-Institute of Microbial Technology (IMTECH), Sector 39-A, Chandigarh, 160036 India

**Keywords:** Binuclear treble clefs, zinc fingers, B-box, ZZ domain, N-end rule, novel fold, U-box

## Abstract

**Background:**

The N-end rule pathway is a part of the ubiquitin–dependent proteolytic system wherein N-recognin proteins recognize the amino terminal degradation signals (N-degrons) of the substrate**.** The type 1 N-degron recognizing UBR-box domain of the eukaryotic Arg/N-end rule pathway is known to possess a novel three-zinc-stabilized heart-shaped fold.

**Results:**

Using sequence and structure analysis we argue that the UBR-box fold emerged from a binuclear RING-like treble clef zinc finger. The RING-like core is preserved in the UBR-box and the metal-chelating motifs display significant sequence and structural similarity to B-box and ZZ domains. UBR-box domains retrieved in our analysis co-occur with a variety of other protein domains, suggestive of its involvement in diverse biological roles. The UBR-box is a unique family of RING-like treble clefs as it displays a distinct circular permutation at the zinc-knuckle of the first zinc-binding site unlike other documented permutations of the RING-like domains which occur at the second zinc-binding site. The circular permutation of the RING-like treble clef scaffold has possibly aided the gain of a novel and relatively deep cleft suited for binding N-degrons. The N- and C-terminal extensions to the circularly permuted RING-like region bind a third zinc ion, which likely provides additional stability to the domain by keeping the two halves of the permuted zinc-knuckle together.

**Conclusions:**

Structural modifications and extensions to the RING-like core have resulted in a novel UBR-box fold, which can recognize and target the type 1 N-degron containing proteins for ubiquitin-mediated proteolysis. The UBR-box appears to have emerged during the expansion of ubiquitin system pathway-related functions in eukaryotes, but is also likely to have other non-N-recognin functions as well.

**Reviewers:**

This article was reviewed by Eugene Koonin, Balaji Santhanam, Kira S. Makarova.

**Electronic supplementary material:**

The online version of this article (doi:10.1186/s13062-015-0066-5) contains supplementary material, which is available to authorized users.

## Background

The N-end rule pathway relates the biological half-life of cellular proteins to the presence of N-terminal destabilizing signals (N-degrons), which upon being recognized by N-recognins, targets the substrate protein for proteolysis [1]. In eukaryotes, two branches of the N-end rule pathway, *viz*., the Arg/N-end rule and the Ac/N-end rule recognize and target different sets of N-degrons [1]. While the Arg/N-end rule pathway recognizes N-terminal arginylated residues and N-terminal basic unmodified residues as N-degrons, the Ac/N-end rule pathway recognizes small uncharged acetylated N-terminal residues [1]. Although, differing in the kind of recognized N-degron and the steps involved in their processing, both branches lead to a common ubiquitin mediated-proteasomal degradation of their targets [1].

**Fig. 1:**
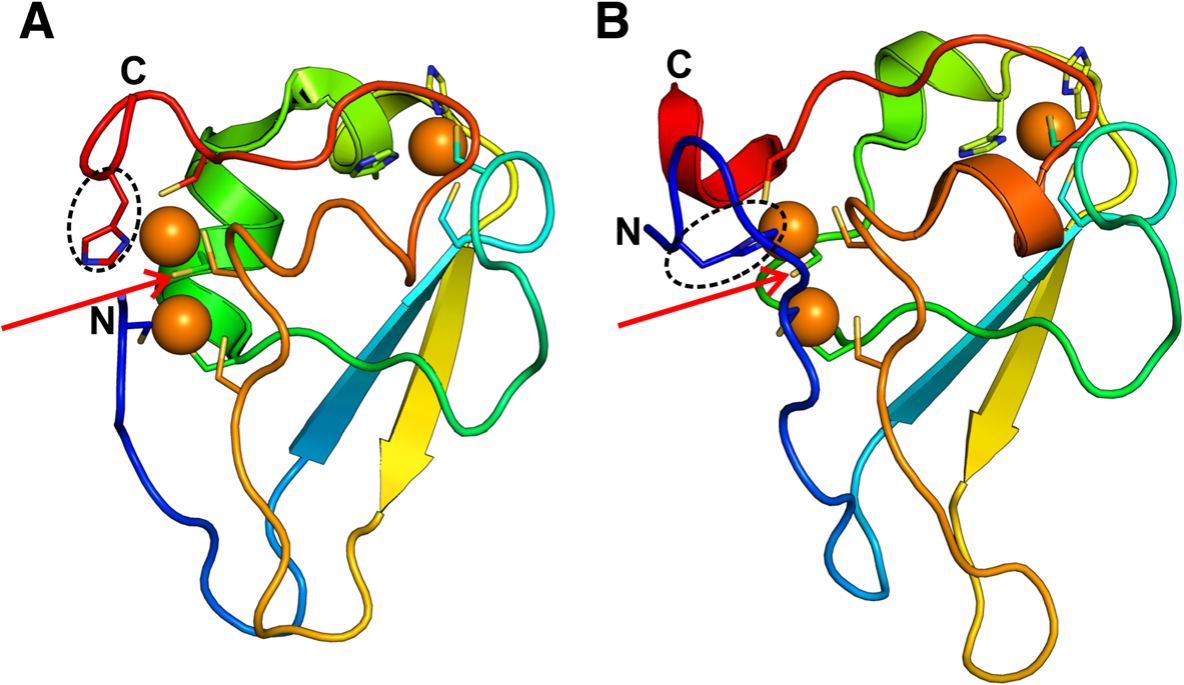
Structure of the UBR-box (**a**) UBR-box from human UBR1 (PDB identifier 3NY1_A) (**b**) UBR-box from *S. cerevisiae* UBR1 (PDB identifier 3NIH_A). The structures have been colored from the N- to C-terminal in a gradient of blue to red. The zinc-chelating residue which displays circular permutation in *S. cerevisiae* UBR-box with respect to the human UBR-box is marked with a dotted circle. The shared metal-chelating residue is indicated by a red arrow

N-recognins of the eukaryotic Arg/N-end rule are distinguished by the presence of a type 1 N-degron recognizing UBR-box domain [1, 2]. The UBR-box is a 70–80 residue domain and is known to be present in at least seven mammalian proteins (UBR1-7) [1–3], two yeast proteins (*Saccharomyces cerevisiae* UBR1 and UBR2) [1, 2] and one plant protein (*Arabidopsis thaliana* PRT6) [2]. The X-ray structures of UBR-box from human UBR1 and UBR2 (PDB identifiers 3NY1_A and 3NY3_A, respectively) [4] and *S. cerevisiae* UBR1 (PDB identifier 3NIJ_A) [5] are available (Fig. 1a,b), both in their apo forms as well as in complex with N-degron containing peptides. The structure of UBR-box has been described as a novel three-zinc-stabilized heart-shaped fold [5]. Human UBR-box is made up of two antiparallel β-strands, two α-helices and two long ordered loops [4]. The *S. cerevisiae* UBR-box [5], though superimposable over its entire length on the human UBR-box lacks the two ordered α-helices and has three short 3_10_-helices instead. Two residues of the UBR-box of human UBR1 (*viz*., V122L and H136R) are reported to be mutated in Johanson-Blizzard syndrome, a recessive genetic disease associated with pancreatic insufficiency, physical malformations and mental retardation [6, 4, 7].

**Fig. 2:**
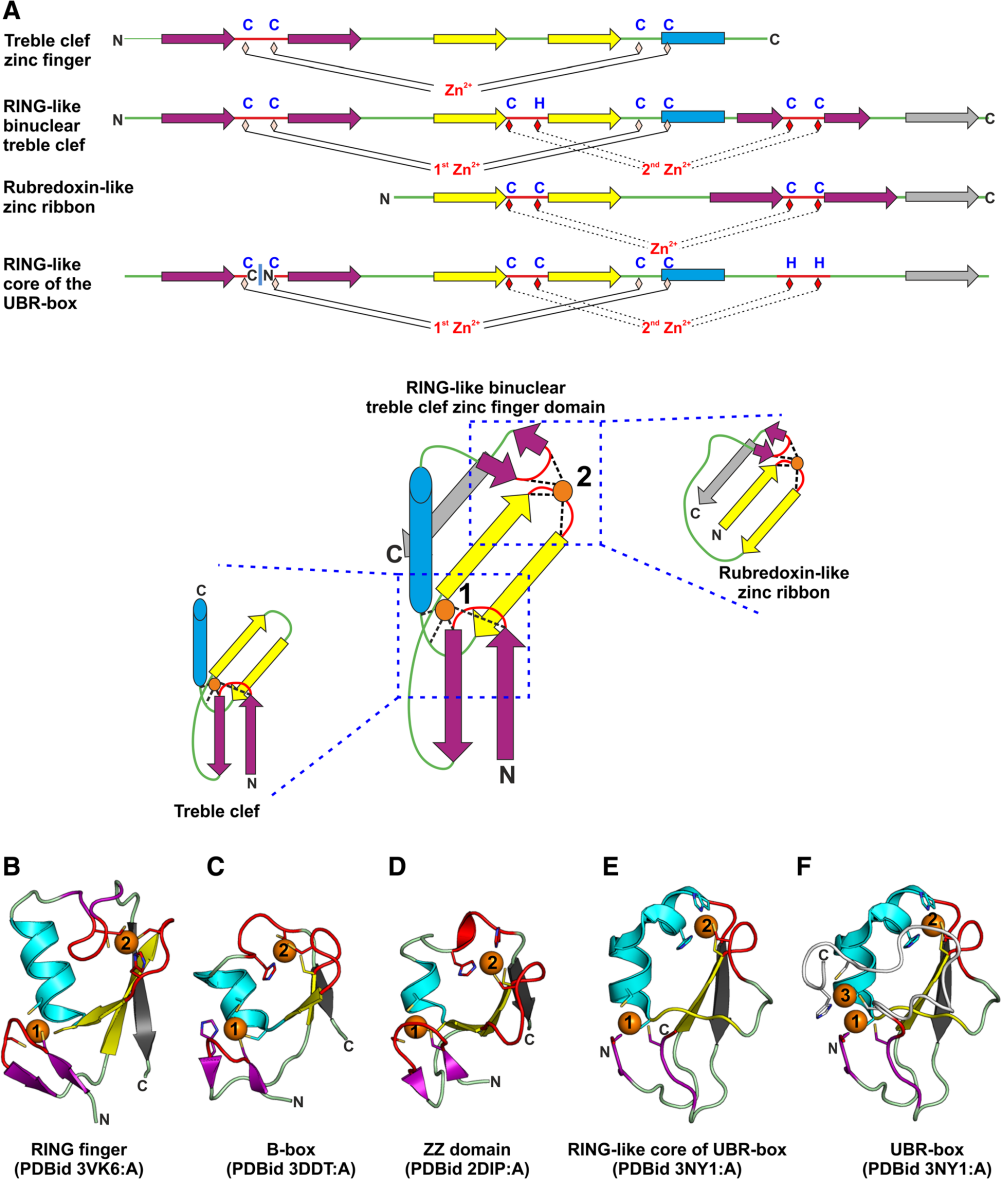
Comparison of the UBR-box with binuclear RING-like treble clef domains (**a**) Arrangement of secondary structure elements in mononuclear treble clef, binuclear RING-like treble clef, zinc ribbon domains and the circularly permuted RING-like core of the UBR-box fold**.** The position of zinc ligands is indicated by diamonds. The most commonly observed metal-chelating aminoacids in the respective domains as per Pfam consensus are shown in blue above the diamonds. For the RING-like binuclear treble clef, the zinc-binding motif of the classical RING family is depicted. However, the motif is variable among different RING-like treble clefs, for example, in the B-box and ZZ domain, the commonly observed metal-chelating motif is CC/HCCCCHH and CCCCCCHH, respectively. The zinc ions have been numbered as per their standard reference for treble clef folds [8, 10]. The zinc ion of the treble clef is numbered ‘1’, the second zinc ion seen in binuclear treble clefs is numbered ‘2’ and the third zinc ion of the UBR-box is numbered ‘3’. β-strands are represented as arrows and α-helices are shown as rectangles/cylinders. The secondary structure elements of mononuclear treble clef have been colored as follows: zinc-knuckle in red, zinc-knuckle containing β-hairpin in purple, primary β-hairpin in yellow and α-helix in cyan. In the binuclear RING-like treble clefs, an additional β-strand is present at the C-terminal which is colored grey. For the rubredoxin-like zinc ribbon region, the primary β-hairpin is colored purple, the secondary β-hairpin in yellow and the zinc-knuckles in red. As indicated, the first and second zinc-binding sites of the RING-like domain structurally resemble a classical mononuclear treble clef and a rubredoxin-like zinc ribbon domain, respectively. (**b**) RING finger domain from E3 ubiquitin-protein ligase Hakai (PDB identifier 3VK6_A) (**c**) B-box from E3 ubiquitin-protein ligase TRIM63 (PDB identifier 3DDT_A) (**d**) ZZ domain from zinc finger SWIM domain-containing protein 2 (PDB identifier 2DIP_A) (**e)** Binuclear RING-like region of the UBR-box (PDB identifier 3NY1_A) (**f**) The full length UBR-box (PDB identifier 3NY1_A) with extensions to the RING-like core, which ligate the third zinc ion, colored in white. The RING-like core scaffold in panels (**b-f**) has been colored similarly. Zinc ions are shown as orange spheres and side chains of zinc-chelating aminoacids are represented as sticks

As the UBR-box is known to be present only in eukaryotes and possesses a novel fold, we were interested in understanding the evolutionary origin of this domain. Here, using sequence and structure-based arguments, we show that the UBR-box is evolutionarily related to the binuclear Really Interesting New Gene (RING)-like treble clef zinc fingers. Classical treble clefs are mononuclear, *i.e.*, they are small polypeptides which fold around a single divalent metal ion (usually zinc) and contain a zinc-knuckle, a primary β-hairpin and an α-helix (Fig. 2a) [8]. A pair of metal-chelating ligands (usually Cys/His) from the zinc-knuckle and a pair from the beginning of the α-helix bind the zinc ion. The RING finger is a binuclear domain with a treble clef fold followed by a small β-hairpin (also called as a ‘squiggle’ [9]) and a C-terminal β-strand (Fig. 2a,b) [8–10]. This C-terminal β-strand forms a three-stranded β-sheet with the primary β-hairpin of the treble clef. The C-terminal extensions to the core of the treble clef and the turn of the primary β-hairpin provide ligands to chelate a second zinc ion (Fig. 2a,b). The tertiary structure of the region chelating the second zinc ion resembles a rubredoxin-like zinc ribbon, with the primary β-hairpin of the treble clef being one of the knuckle-containing β-hairpins of the zinc ribbon [8] (Fig. 2a).

Many binuclear treble clefs such as the B-box, ZZ, Zf-UBP, TFIIH-p44 and C1-type zinc fingers share two characteristic features with the RING finger. First, these domains contain a three-stranded β-sheet comprising of the primary β-hairpin and the C-terminal β-strand [9]. Second, the position of the last metal-chelating residue is invariably at the beginning of the C-terminal β-strand [9]. Based on the presence of these features, the aforementioned binuclear treble clefs are suggested to comprise a monophyletic group [9], which we refer to as the binuclear RING-like treble clef domains. RING finger domains from a number of proteins have been shown to function as E3 ubiquitin ligases wherein they mediate protein-protein interactions with E2 ubiquitin ligases [11]. Besides the well acknowledged role of RING fingers in the ubiquitination pathway, the B-box and the Zf-UBP are also known to function as E4 ubiquitin ligases [12] and as ubiquitin-binding modules in the aggrosome pathway [13], respectively. Our analysis suggests that the UBR-box domain is yet another novel variant of the RING-like treble clef which functions in the ubiquitin system pathway.

## Results and discussion

The UBR-box may be regarded as a novel three zinc-stabilized fold [4, 5], but a closer inspection reveals striking resemblances to the B-box (Fig. 2c) and ZZ domains (Fig. 2d). On comparison of the structures of the UBR-box with representatives of the B-box, ZZ domain and RING finger (Fig. 2b-e), we observe that the UBR-box core is related by a circular permutation to the RING-like treble clef domain (Fig. 2e) with additional N- and C-terminal extensions that bind a third zinc ion (Fig. 2f). The UBR-box lacks a well-defined second β-strand in the primary β-hairpin of the treble clef and instead harbors a loop at this position. This loop hydrogen bonds with the first β-strand near the first-turn of the α-helix, thus, forming a short region characteristic of the primary β-hairpin of treble clefs (PDB identifiers 3NIT_A, 3NY1_B). Apart from these minor structural changes, the UBR-box has all characteristic features of the RING-like treble clefs [9].

**Fig. 3:**
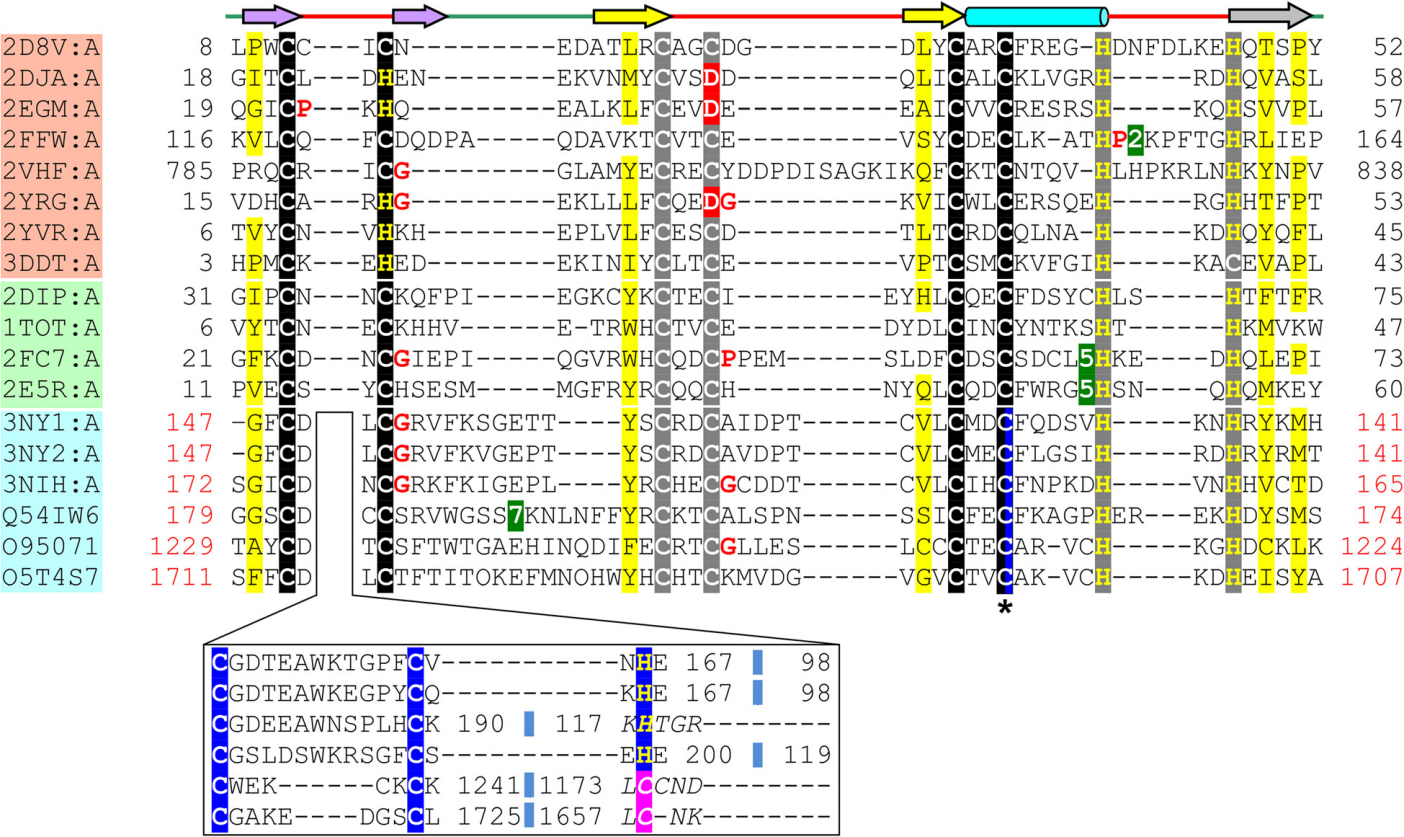
Structure based multiple sequence alignment of B-box, ZZ domain and UBR-box. PDB/UniProt identifier, start and end aminoacid numbers are indicated for each sequence. Identifiers of the representative sequences of the B-box are highlighted in peach, ZZ domain in light green and UBR-box in light blue. Secondary structure diagram is depicted above the alignment. The zinc-binding residues (Cys/His) of the first zinc binding site of the binuclear treble clef have been highlighted in black, those of the second zinc binding site in grey, those of the third zinc binding site (for the UBR-box) in dark blue and other aminoacids at equivalent position in red. Residues that may potentially serve as metal-chelating ligands are highlighted in pink. The shared ligand in the UBR-box is indicated by an asterisk (*) and highlighted in black and blue. The sequence region in between the circularly permuted zinc-knuckle of the UBR-box, which is not present in the B-box and the ZZ domain, is shown in a separate box under the alignment of the common binuclear RING-like regions. Regions of circular permutation are separated by a small blue colored box and sequence numbers of the regions around the circular permutation are colored in red. Regions where the structures are not superimposable are shown in italics. Small aminoacids (Gly, Pro) in the vicinity of the zinc-binding ligands are colored red. Uncharged residues (all aminoacids except Asp, Glu, Lys and Arg) in mostly hydrophobic sites are highlighted yellow. Long insertions are not shown and the number of omitted residues is boxed in green

Sequence similarity searches initiated with the sequences of the complete UBR-box, and independently of the region corresponding to the binuclear RING-like region, are able to retrieve several B-box and ZZ domains as statistically-significant matches. For example, a HHpred [14] search initiated with the UBR-box of *S. cerevisiae* UBR1 (PDB identifier 3NIH_A) was able to find matches to the Pfam B-box zinc finger family (PF00643, E-value = 0.0015), B-box domain of tripartite motif-containing protein 5 (PDB identifier 2YRG_A, E-value = 0.017) and to the Pfam ZZ zinc finger family (PF00569, E-value = 0.015) and ZZ domain of ZZZ3 protein (PDB identifier 2FC7_A, E-value = 0.048). Similarly, FFAS [15] search initiated with UBR-box of *S. cerevisiae* UBR1 (PDB identifier 3NIH_A) retrieved B-box of Midline-2 (PDB identifier 2DJA_A, Score = −9.74) and ZZ domain of zinc finger SWIM domain-containing protein 2 (PDB identifier 2DIP_A, Score = −9.20) as top-scoring matches. A weak resemblance of a part of the UBR-box motif to the ZZ domain of PRT1 has also been noted previously [3]. Additional, PSI-BLAST [16] and JackHMMER [17] searches were initiated with representatives of B-box, ZZ and UBR-box domains to retrieve homologs from the PDB and UniProtKB [18] database. A structure based multiple sequence alignment (MSA) of the B-box, ZZ domain and UBR-box (Fig. 3) reveals a good alignment of all zinc-chelating residues.

The sequences of the UBR-box classified in Pfam (PF02207) and those obtained by the JackHMMER search were analyzed for the presence of co-occurring domains and their distribution across eukaryotes (see Additional file 1 for details). We observe a widespread distribution of the UBR-box and their co-occurrence with a variety of other domains (Additional file 2: Figure S1). We could detect the UBR-box domain in Amoebozoa, Euglenozoa, Diplomonadida, Choanoflagellida, Parabasalia, Heterolobosea, Stramenopiles, Alveolata, Haptophyceae, Ichthyosporea, Fungi, Viridaeplantae and Metazoa. Our analysis suggests that in a majority of proteins, the UBR-box is the only domain identified by automated searches. The commonly co-occurring domains with the UBR-box include the RING, HECT, ZZ, PHD, ClpS and F-box domains. In some of the retrieved sequences, domains such as protein kinase (PF00069), methyltransferase (PF10294, PF05050), zf-RanBP (PF00641), telomerase RNA binding domain (PF12009), DNA polymerase processivity factor (PF02916), pectate lyase (PF12708), concanavalin A-like lectin/glucanases (PF13385), siroheme biosynthesis protein (PF14824, PF14823), oxysterol-binding protein (PF01237), etc., are also present adjacent to the UBR-box domain (Additional file 2: Figure S1**)**.

Previous reports have shown that UBR-box containing proteins function in diverse biological processes [2, 19, 20] such as degradation of misfolded proteins [21], chromosome segregation [22], meiosis [23, 24], spermiogenesis [25], neurogenesis [26], apoptosis [27], cardiovascular development [26], sensing of heme [28], oxygen [29], nitric oxide [30], and short peptides [31], quorum sensing [32], the regulation of peptide import [33, 34], regulation of pancreatic and brain function and development [6], and senescence, germination and hypoxia in plants [35, 36]. The UBR-box domain is referred to as a structural scaffold with varying binding specificities [37] as not all the UBR-box domains interact with N-degrons [1–3] and may bind other moieties which are not necessarily substrates for ubiquitination. For example, binding of short peptides to the UBR-box domain of *S. cerevisiae* UBR1 is known to allosterically regulate the activity of adjacent domains [31, 34]. Currently, the biological function and interacting partners for most non-N-recognin UBR-box domains have not been experimentally demonstrated. A recent report reveals that the UBR-box domain in *Drosophila* non-N-recognin UBR3 can bind and regulate the activity of *Drosophila* inhibitor of apoptosis protein 1 (DIAP1) [38]. The large repertoire of co-occurring domains (Additional file 2: Figure S1**)** identified in our analysis is suggestive of diverse biological roles for the UBR-box.

The UBR-box domain is not classified by SCOP [39] or CATH [40], but has been grouped under the B-box zinc-binding domain-like X-group of ECOD [41] based on structural similarity (ECOD identifier e3ny1A1). Dali structure similarity searches [42] initiated using the human or *S. cerevisiae* UBR-box against the PDB did not find matches to any other proteins as reported earlier [4, 5]. However, TopSearch [43], could identify several mononuclear and binuclear treble clefs, including B-box and ZZ domain, as matches to the structure of UBR-box (PDB identifier 3NY1_A). The best match is that of the mononuclear treble clef domain of Poly(ADP-ribose) polymerase 1 (PARP1; PDB identifier 4AV1_A) with a similarity score of 45.1 and a RMSD of 2.3 Å over an alignment length of 50 C_α_ atoms with a single permutation at the zinc-knuckle. Interestingly, TopSearch [43] could identify the exact site of circular permutation while superimposing the UBR-box with other treble clef domains. Automated and manual pairwise structural superimposition of the UBR-box with B-box and ZZ domains also reveal structural similarity among these treble clefs and they could be aligned over the full length of their corresponding binuclear RING-like regions upon assuming circular permutation at the zinc-knuckle. For example, using TM-Align [44], the circularly permuted RING-like region of UBR-box (PDB identifier 3NY1_A) could be superimposed on the B-box domain (PDB identifier 3DDT_A) with an RMSD of 2.7 Å over 38 C_α_ atoms with a TM-Align score of 0.44 (normalized over the length of the B-box). The circularly permuted binuclear RING-like region of UBR-box (PDB identifier 3NY1_A) and the B-box domain (PDB identifier 3DDT_A) could be manually superimposed using the pair fitting command of PyMOL with an RMSD of 2.46 Å over 40 pairs of C_α_ atoms (Additional file 3: Figure S2). Thus, the structural and sequence similarities discussed above suggest an evolutionary connection between the UBR-box and binuclear RING-like treble clefs.

The third zinc ion of the UBR-box, chelated by three ligands from the C-terminal extension in human UBR1 and a shared ligand with the first zinc ion, is important for the N-degron-binding function [4, 5] and perhaps provides additional structural stabilization to the circularly permuted zinc-knuckle. Circular permutations to the core of a treble clef fold are rare unlike their relatively common occurrence in the zinc ribbon fold [10, 45], and a previously documented example of circularly permuted treble clef is that of the C-terminal domain of prolyl-tRNA synthetase (PDB identifier 1H4Q_A) [10, 46]. However, we note the presence of permuted treble clefs in other structures such as the triquetra knot containing protein Rds3p (PDB identifier 2K0A_A; ECOD identifiers e2k0aA1, e2k0aA2, e2k0aA3) (Additional file 4: Figure S3). Circular permutations to the RING-like treble clefs have been observed in C1 and TFIIH-p44 zinc finger domains [10, 8] (Additional file 5: Figure S4) but in these structures the permutation occurs at the zinc ribbon-like region, which harbors the second zinc-binding site. Circular permutation at the first zinc-binding site of binuclear RING-like treble clefs is unique to the UBR-box, though a similar permutation is seen in one other mononuclear treble clef, *viz*., the Rds3p, which has three treble clef motifs of which two are circularly permuted (PDB identifier 2K0A_A) (Additional file 4: Figure S3) [47]. The third zinc ion of the UBR-box, which shares a metal-chelating residue with the first zinc ion of the treble clef, forming a two-metal ion cluster is also not unprecedented. Such two-metal ion clusters are observed in other treble clefs, such as the RING finger of RAG1 (PDB identifier 1RMD_A) and FYVE/PHD zinc finger from the C-terminal region of E3 ubiquitin-protein ligase CHFR (PDB identifier 2XOC_A). Thus, we observe that the structural modifications to the treble clef fold of the UBR-box have precedents and these further corroborate the relationships between the UBR-box and RING-like domains.

Based on sequence and structural similarities of their binuclear regions, we hypothesize that the UBR-box plausibly emerged via circular permutation of a RING-like treble clef scaffold. The circular permutation likely resulted in the splitting of the treble clef’s zinc-knuckle and joining of the C-terminal β-strand to the first-half of the split knuckle. This in turn, resulted in the formation of a relatively deep-binding cleft on which the N-degron-recognizing and -binding functions likely emerged. The extensions to the RING-like core perhaps developed subsequently, to provide additional structural stability to the permuted zinc-knuckle. These extensions are also important for function as they harbor residues that help form an acidic cleft to bind the basic type 1 N-degrons [4, 5]. A comparison of the UBR-box from human and *S. cerevisiae* UBR1 revealed that one of the ligands for the third zinc ion (His166 in human UBR1, PDB identifier 3NY1_A and His118 in *S. cerevisiae* UBR1, PDB identifier 3NIH_A) while being present at an equivalent spatial location, is circularly permuted with respect to its position in the sequence (Fig. 1a,b). In the human UBR-box, this His is present on the C-terminal extension of the UBR-box, whereas in the *S. cerevisiae* protein, it is located at the N-terminal region. It has been noted previously that all zinc-chelating residues with the exception of this His are conserved among human UBR proteins [4]. However, we observe the presence of other metal-chelating aminoacids in the N-terminal extensions of the RING-like core of the UBR-box, which are positioned equivalent to the *S. cerevisiae* N-terminal His in the sequence and may help chelate the third zinc ion (for example, Cys1174 in human UBR5, UniProt ID: O95071, Cys1658 in human UBR4, UniProt ID: Q5T4S7 in Fig. 3).

It is believed that metal chelation aided the emergence of new protein folds, concomitant with the advent and expansion of novel functions in eukaryotes [48]. Also, it is known that many binuclear treble clefs were already present before the emergence of the Last Eukaryotic Common Ancestor (LECA) and at least one binuclear treble clef was present in the Last Universal Common Ancestor (LUCA) [9]. It has been suggested that some of the binuclear RING-like treble clefs present in the LECA diverged into new superfamilies in eukaryotes and gained distinct biochemical functions [9, 48]. The B-box is one such binuclear RING-like treble clef, which is present in both prokaryotes and eukaryotes where it is involved in the folding, stability and proteolysis-related functions of membrane proteins, and in ubiquitin system pathway-related functions, respectively [9]. The ZZ domain on the other hand is present only in eukaryotes [49, 50] where it is proposed to play a scaffolding role and mediate protein-protein interactions [50]. The B-box and the ZZ domain share sequence and structural similarities over the full length of the binuclear treble clef domain [51] and are likely related in evolution. The UBR-box appears to have diverged only recently from the binuclear RING-like treble clef group given that it is present only in eukaryotes [49, 2, 1] where it functions in the Arg/N-end rule pathway [1]. A recent emergence of the UBR-box from RING-like treble clefs is also supported by similar patterns of emergence of other components of the Arg/N-end rule pathway [52]. For example, Arg-conjugating enzymes that lead to the addition of primary destabilizing N-terminal Arg on secondary destabilizing N-terminal residues are likewise confined to eukaryotes [52].

Intriguingly, type 2 N-degron recognizing PRT1 protein from *A. thaliana* is proposed to interact with the aromatic N-terminal residue of the type 2 N-degron via its ZZ domain [53]. Although not very well characterized, ZZ-domains are suggested to have two surfaces, a hydrophobic patch and an acidic region, which could likely interact with other proteins [50]. Some of the conserved hydrophilic and hydrophobic aminoacids are at a similar structural location as the region utilized by the UBR-box to interact and bind its substrate. A comprehensive survey of the various binding surfaces used by mononuclear [8] and binuclear treble clefs has been reported earlier [9]. However, the mode of binding the N-degron peptide in a relatively deep cleft within the treble clef domain, where the peptide forms antiparallel β-sheet interaction [5] with the zinc-knuckle containing β-strand, is observed only in the UBR-box. The backbone atoms of the first three residues and the side chains of the first two residues of the N-degron contact the UBR-box [5, 4]. The N-terminal basic residue is accommodated in an acidic cleft formed by residues contributed primarily by the first-half of the split zinc-knuckle and the C-terminal extension [5]. Additionally, residues on the primary β-hairpin and the first-half of the split zinc-knuckle are involved in hydrogen bonds and hydrophobic interactions with the N-degrons [5]. A similar binding pocket is seen in the PARP1 mononuclear treble clef (PDB identifier 4AV1_A), which recognizes and binds dsDNA [54] (Additional file 6: Figure S5). In PARP1, an additional N-terminal β-strand forms a three-stranded antiparallel β-sheet with the primary β-hairpin of the treble clef and connects to the zinc-knuckle containing β-hairpin, forming a topologically similar connection to that seen in the UBR-box.

## Conclusions

We show that the UBR-box is a novel member of the RING-like treble clef fold and is related to the B-box and ZZ domains. The UBR-box is present only in eukaryotes and appears to have emerged during the expansion of ubiquitin system pathway-related functions [48, 9]. However, its function is not merely confined to recognition of N-recognins and it may serve diverse biological roles by mediating interactions with other proteins. The structural modifications including the circular permutation and the functionally important extensions to the RING-like core has resulted in the emergence of a novel UBR-box fold capable of binding type 1 N-degrons. The relationship of RING-like domains to the UBR-box, illustrates the versatility of the treble clef scaffold upon which novel functions may emerge on different regions of the domain [8–10, 55].

## Methods

### Structure based methods

Structures of the human and *S. cerevisiae* UBR-box (PDB identifiers 3NY1, 3NY2, 3NY3, 3NIH, 3NII, 3NIJ, 3NIK, 3NIL, 3NIM, 3NIN, 3NIS and 3NIT) and other treble clef domains were retrieved from the PDB. Dali [42], TopSearch [56], TM-align [44] and Fr-TM-align [57] tools were used to evaluate structural similarity of the UBR-box with other proteins. The structures were visualized, compared and manually superimposed in the molecular visualization program, PyMOL. Manual structural superimposition was performed by defining the equivalent regions using the pair fitting command of PyMOL.

### Sequence based methods

Sequence similarity searches initiated with the UBR-box domain were performed using iterative PSI-BLAST (against PDB database of Feb 28, 2015, Number of letters: 18,460,040, Number of sequences: 75,710; E-value threshold of 0.001) [16], JackHMMER program from the HMMER3 package (against NCBI non-redundant (NR) version 2014-06-17 and UniProtKB version 2014-06-17; E-value threshold of 0.01) [17], FFAS server [15] (against regularly updated PDB, Pfam and SCOP databases) and HHpred server [14] (against PDB70_ 24Jan15 and PfamA_27.0, using MSA generation method HHblits run for 5 iterations, E-value threshold of 0.001). MSAs of the treble clef domains were made using the ClustalW program [58] within the BioEdit software package (version 7.2.2) with default parameters [59]. Thereafter, structure based manual adjustment of the MSAs was done.

## Reviewers' comments

### Review #1: Eugene V. Koonin, Senior Investigator, National Center for Biotechnology Information, National Institutes of Health, Bethesda, MD USA

*Report form: Kaur and Subramanian examine the structure of the UBR box and show that it is a circular permutation of the binuclear treble clef Zn finger rather than a truly novel Zn-binding fold. To the best of my understanding, this is a valid relationship and as such, quite a useful observation. In my opinion, the authors miss out on phylogenomic analysis of the UBR box. I think it would have been quite informative to identify UBR box-containing proteins across the entire eukaryotic diversity and reconstruct the scenario of evolution. Is N-degron recognition the only function of the UBR box or are there additional exaptations? This question could be addressed through careful analysis of the domain architectures of the UBR box-containing proteins. As it stands, the manuscript reads more like a Discovery Note although the format is that of a research article. Expansion along the above lines could rectify this mismatch between content and form.*

*The manuscript is not carefully written or edited, careful editing is a must.*

*Quality of written English: Needs some language corrections before being published*

#### Response to the Report:

We have now analysed and reconstructed phylogeny for UBR-box domains across all eukaryotes. A phylogenetic tree of representative UBR-box domains is included as an additional figure. Domains co-occurring with the UBR-box are provided besides the branch labels. Our analysis, similar to earlier studies, reveals that the UBR-box co-occurs with a diverse set of protein domains. Most domain architectures are suggestive of a likely involvement of the UBR-box containing proteins in ubiquitin-related pathways. However, it is also known that many UBR-box containing proteins lack the N-recognin function and do not bind N-degrons. In such scenarios, the most plausible function of the UBR-box may be in mediating protein-protein interactions, as discussed in the manuscript.

### Review #2: Dr. Balaji Santhanam, Senior Investigator Scientist, MRC Laboratory of Molecular Biology, Francis Crick Avenue, Cambridge Biomedical Campus, Cambridge, CB2 0QH, UK

*Report form: In this manuscript the authors argue, based on sequence and structure analyses, for the relationship between UBR-box and binuclear RING-like treble clef zinc fingers such as RING, B-BOX, ZZ, PHD and MYND fingers. The authors provide convincing arguments for how UBR-box is a unique circularly permuted version of binuclear treble clef zinc finger and indeed related to other binuclear treble clef fingers. Their proposal appears scientifically sound and convincing. However, I have a few comments listed below:*

1. *There seems to some intertwined arguments for relationship between UBR-box and binuclear treble clef fingers. These include systematic structural searches, followed by sequence searches and also manual analyses (Line # 107 to 152). The organization and writing of this part is confounding the understanding of the strategies to link UBR to binuclear treble clef fingers.*
2. *I found it rather strange that the authors narrate their structural analyses first and discussed the sequence analyses later. Sequence searches clearly show clear link between UBR and ZZ, B-Box (at least for significant parts of the domains). This also possibly adds to the confusion stated above. I would have naturally expected sequence searches to be the first straightforward way to get an evolutionary link between domains. Hence, has to be the first one to be discussed here.*

#### In response to 1 and 2:

The relationship between UBR-box and RING-like domains has been established using a combination of statistically-supported sequence and structural similarities. Interestingly, the similarity of the UBR-box to RING-like domains can be made only upon assuming a circular permutation of the structures. The evidence for the circular permutation is primarily based on structural similarity, i.e., assuming this permutation allows us to superpose the entire core of the RING-like domain on the UBR-box. While sequence similarity statistics between the UBR-box and RING-like domains is convincing, however none of the automated tools can recapitulate the alignment over the entire length of the RING-like core because of the circular permutation and remote similarity. Hence, the structural similarity to RING-like domains was discussed first followed by sequence similarity which helps us to establish the evolutionary link between UBR-box and RING-like domains. We have nevertheless taken into consideration the comments of the reviewer and discuss sequence similarity before the structural evidence in the revised manuscript.

3. *There was no detailed discussion on their observation of evolutionary link between ZZ, B-box and UBR later as well.*
4. *Given there is strong sequence/structure relationship between UBR-box and ZZ and B-box, I was expecting an evolutionary model similar to the Ref. 9 (Burroughs et al.). Ref. 9 suggests that FYVE finger is likely to have evolved from a mononuclear version through LIM like intermediate through circular permutations (CP). Is it possible that UBR-box evolved by CP of either ZZ or B-box precursor? Did the authors look for UBR-like proteins in prokaryotes?*

#### In response to 3 and 4,

In the current manuscript we provide a detailed discussion about the relationships between the ZZ, B-box and UBR-box domains. It must be noted that these domains are distantly related. Given the evidence from sequence and structural similarities, we can speculate that the UBR-box emerged via circular permutation of a RING-like domain such as the ZZ or B-box. However, there is no clear cut evidence to pinpoint to the exact precursor. Our sequence similarity searches do not retrieve any UBR-box sequences, which are not circularly permuted with respect to the RING-like treble clefs. Also, we are not able to identify any UBR-like proteins in prokaryotes at this time.

5. *As articulated in Ref. 9 binuclear treble clef fingers have independently or by convergence acquired Ub-related functions. UBR seems to be no exception; do the authors have any comment?*

Yes, it is possible that the UBR-box may have independently acquired Ub-related function. However, it must be noted that in the type 2 N-degron recognizing PRT1 protein from *A. thaliana*, a ZZ domain is suggested to interact with the N-terminal destabilizing residue (please refer to Stary *et al.*, 2003 [53]). Thus, it is possible that a RING-like scaffold with a generic function of interacting with polypeptides was the ancestor of the ZZ and UBR-box.

6. *Structural search strategy appears less systematic than the sequence analyses. I understand that this is possibly due to DALI searches did not yield any significant hits. Did the authors try running DALI locally on an expanded database of structures?*

Our structural search strategy primarily involves the TopSearch tool to scan the entire PDB for structures similar to the UBR-box. Dali and TM-Align were used for reconfirming the structural matches. In our opinion, TopSearch is presently one of the best programs to detect structural similarities between proteins related by circular permutations. Our manual structural analysis also support the results obtained by TopSearch. Our primary purpose in this manuscript was to determine if the UBR-box is indeed a new fold or if it is evolutionarily related to any other well classified zinc finger domain. So we did not deem it necessary to run Dali locally on an expanded database of structures.

7. *It is rather strange to me that structural searches using TopSearch yielded best hit to PARP1 and not to ZZ or B-box as suggested by sequence searches. Do the authors have any explanation?*

TopSearch perhaps finds highest similarity to PARP1 because of an extensive structural similarity of the protein domains, especially in the regions involved in ligand binding. We notice that the UBR-box and the treble clef domain of PARP1 have a similar twist in their respective primary β-hairpins. Also, the additional β-strand which forms a three stranded β-sheet with the primary β-hairpins in both the proteins is similarly extended into the knuckle-containing β-strand and helps sculpt the respective ligand binding regions.

### Review #3: Kira S. Makarova, National Center for Biotechnology Information, National Institutes of Health, Bethesda, MD USA

*Report form*: *The paper presents a relatively straightforward sequence and structure comparative analysis of several families of RING treble clef zinc finger domain. Based on this analysis authors hypothesized that UBR box domain originated though a circular permutation of the ancestral RING-like domain. This hypothesis considering presented data appears plausible and the text and figures are generally clear. I don’t have any significant criticism for this work. The only problem I have with the paper is its length and relevance of some parts of the text and some Figures. I believe that the arguments in support of this hypothesis could be described in a text half of the current size and rather in a format of a Discovery Note paper than the original research paper.*

*Below I have a list of relatively minor suggestions how to improve the clarity of the paper and what parts could be dispensable in the main text.*

1. *For a better evolutionary perspective and understanding the UBR-box family I’d recommend to start Result and Discussion with a brief description of diversity of this domain in different eukaryotic lineage. It can be described together with results of other sequence similarity searches and the Fig. four should be moved forward accordingly. Furthermore the details of sequence similarity searches could be moved to Additional material and only essence of that searches mentioned in the text. In the Fig. four organisms to which aligned sequences belong should be indicated and more UBR-box sequences from different eukaryotic lineages should be shown in the multiple alignment accordingly. Otherwise it not clear when the permutation happened during the evolution of this domain.*
  We have taken all suggestions of the reviewer to improve the manuscript. We have added details relating to the distribution of the UBR-domain in eukaryotes in the Results and discussion section.
2. *There is no need to mentioned other cases of the permuted treble clef zinc finger domain, especially if they are not described as such in the literature (for example, domain in CasA protein of CRISPR-Cas system and respective Additional material). So if authors find it interesting then they should write a different a paper showing this explicitly or a review describing this phenomenon in general.*
  Examples of circular permutations in the treble clef domain are rather rare. We have only mentioned well documented cases of circular permutations with the exception of the CRISPR system Cascade subunit CasA. The purpose of this section was to illustrate that the circular permutation observed in UBR-box is not unprecedented and a similar permutation is observed in the third treble clef domain of the triquetra knot containing protein Rds3p. For this reason, we would like to retain this section.
3. *On the Fig.*2*a please indicate respective cysteines and histidines for Zn ligands. Indicate the fact that pink strands are actually not structured in UBR-box structures.*
  The zinc-chelating residues have not been labelled in Fig. 2a as there isn’t absolute conservation of the aminoacids involved in metal-chelation. Cysteines and histidines are the most commonly observed residues which chelate the zinc ions as is evident from the MSA.
  The representation of the UBR-box in Fig. 2a is shown with the β-strands colored in pink to indicate equivalencies among the various RING-like domains. Although, the pink colored β-strands of the UBR-box are not structured in the apo-form, at least one of these takes up a β-strand conformation in the holo-form. These structural details are also mentioned in the text.
4. *Comparison with PARP1 mononuclear treble clef does not add anything to the story, as the Fig. 5. It can be either moved to the supplement or omitted entirely. The same applies to the Fig.*3*.*
  We have moved these figures to the Additional files section as suggested by the reviewer. However, we would like to retain the text which compares PARP1 and the UBR-box. PARP1 is among the best scoring hits in TopSearch and binds DNA in a similar region to that used by UBR-box to bind the N-degron peptide. Analysis of binding sites in all treble clef domains suggest that this region is unique to these two proteins and we feel it is appropriate to discuss this in the context of the UBR-box.

## Reviewers’ comments – second round of review

### Reviewer #1 and #2 had no additional comments.

#### Reviewer #3

*Report form: The paper presents a relatively straightforward sequence and structure comparative analysis of several families of RING treble clef zinc finger domain. Based on this analysis authors hypothesized that UBR box domain originated though a circular permutation of the ancestral RING-like domain. This hypothesis considering presented data appears plausible and the text and figures are generally clear. I don’t have any significant criticism for this work.*

*After the first round of revision the clarity of the paper improved, although there are still some parts of the paper that are not explained in sufficient details and are not directly relevant for the main point of the paper. As I mentioned before this concerns circular permutations in the treble clef domain of CasA protein and the triquetra knot in Rds3p protein which were “noticed” but not elaborated by the authors (now both are shown in the supplement). Since no analysis of these cases were provided this leaves a reader with the only option to believe that the authors are correct in their interpretations.*

Taking into consideration the concerns of the reviewer, we have removed the text and figure illustrating the circularly permuted treble clef motif which we observed in the CasA protein. However, we retain the prolyl-tRNA synthetase and Rds3p examples as these treble clef motifs have already been documented and discussed in previously available literature [10, 47].

*I also still have an issue with the authors’ response to my request to indicate aminoacids involved in Zn binding on the Fig.*2a* in order to have a better understanding of the amino acid signatures of the 1st and 2nd Zn binding motif. I expect that each family shown in the Fig.*2a* has a typical signature (consensus) for Zn binding ligands. These residues don’t have to be 100 % conserved. Showing this consensus is the same as showing a generalized representation of the secondary structure elements for the respective domains (which is shown in the Fig.*2a*), although some proteins could have deviations from this typical organization.*

We have added the metal-chelating aminoacids for the domains as per Pfam consensus in Fig. 2a and also provide a short description in the figure legend. The side chains of the metal-chelating residues are also shown as sticks for each of the domains in their tertiary structures in Fig. 2b-f.

## Additional files

Additional file 1:
**Methodology used to make Additional file** 2 **: Figure S1.**
Additional file 2: Figure S1. Phylogenetic relationships between UBR-box domains. The figure depicts the bootstrap consensus tree for UBR-box domains from various eukaryotic taxa. Branch labels denote the UniProt ID/gi and organism name separated by ‘|’. The coloring scheme used to highlight proteins from different taxa is indicated. The domain architectures for the proteins are shown besides the branch labels. Pfam domains are represented by different shapes. Additional file 3: Figure S2. Stereo diagram of the binuclear RING-like region of UBR-box and B-box. The stereo structures of the binuclear RING-like region of UBR-box from human UBR1 (PDB identifier 3NY1_A, colored red) and the B-box from E3 ubiquitin-protein ligase TRIM63 (PDB identifier 3DDT_A, colored blue) are shown. The structures were manually superimposed in PyMOL using the pair fitting command and translated thereafter. The backbone C_α_ chain trace is shown for both domains. The zinc ions are shown as spheres and zinc-chelating residues are shown as lines. Additional file 4: Figure S3. Circularly permuted treble clef zinc finger domains. **(A)** Prolyl-tRNA synthetase C-terminal domain (PDB identifier 1H4Q_A) **(B-D)** Three treble clef motifs (TC_1, TC_2, TC_3) of Rds3p protein (PDB identifier 2K0A_A). TC_1 **(B**) and TC_3 **(D)** of Rds3p are permuted with respect to the classical treble clef seen in TC_2 **(C)** of Rds3p. The permutation seen in TC_3 is at the same position as that of the UBR-box. Coloring scheme for **(A-D)** follows Figure 2. **(E)** Structure-based multiple sequence alignment of the treble clef domains in **(A-D)**. Coloring scheme for MSA follows Figure 3. Additional file 5: Figure S4. Circular permutations in binuclear RING-like treble clef domains. **(A)** Binuclear RING-like region of the UBR-box (PDB identifier 3NY1_A) **(B)** RING finger domain of E3 ubiquitin-protein ligase Hakai (PDB identifier 3VK6_A) **(C)** Zinc finger domain of TFIIH-p44 (PDB identifier 1Z60_A) **(D)** C1 domain of Protein kinase C (PDB identifier 1PTR_A) **(E)** DC1 domain of PDI-like hypothetical protein At1g60420 (PDB identifier 1V5N_A). Coloring scheme for **(A-E)** follows Figure 2. **(F)** Structure-based multiple sequence alignment of the binuclear RING-like treble clef domains in **(A-E)**. Coloring scheme for MSA follows Figure 3. Additional file 6: Figure S5. Stereo diagram of ligand binding sites in UBR-box and PARP1. **(A)** The N-degron bound UBR-box from yeast UBR1 (PDB identifier 3NIN) **(B)** The dsDNA bound treble clef of PARP1 (PDB identifier 4AV1). The secondary structure elements of the treble clef domains are colored identically. Coloring scheme follows Figure 2. The bound peptide/DNA is colored dark blue.

## Abbreviations

UBR: ubiquitin ligase N-recognin
RING: Really Interesting New Gene
PDB: Protein Data Bank
SCOP: Structural Classification of Proteins
CATH: Class, Architecture, Topology, Homology
ECOD: Evolutionary Classification of protein Domains
MSA: Multiple Sequence Alignment
LECA: Last Eukaryotic Common Ancestor
LUCA: Last Universal Common Ancestor
PARP: Poly (ADP-ribose) polymerase
HECT: Homologous to the E6-AP Carboxyl Terminus
