# Supplementary material for: The UBR-box and its relationship to binuclear RING-like treble clef zinc fingers
Source: Biol Direct. 2015 Jul 17;10:36. doi: 10.1186/s13062-015-0066-5 (PMC4506424; doi:10.1186/s13062-015-0066-5)
Supplement: Additional file 1: — Methodology used to make Additional file 2 : Figure S1. [file 13062_2015_66_MOESM1_ESM.docx]

**The UBR-box and its relationship to binuclear RING-like treble clef zinc fingers**

Gurmeet Kaur, Srikrishna Subramanian

**Additional Figure 1**

**Methodology:**

The domain architectures of proteins containing a UBR-box domain as classified in Pfam version 27.0 (PF02207) were analysed. Additionally, distinct domain architectures obtained in iterative JackHMMER search with UBR-box domain of *S. cerevisiae* UBR1 (PDB identifier 3NIH) were also analysed. The sequences of UBR-box domains classified in Pfam (PF02207) were retrieved and clustered at 70% sequence identity and 90% sequence coverage using cd-hit [[1](#_ENREF_1)]. The sequences for the UBR-box domains from proteins with distinct domain architectures that were not present in the sequences obtained after clustering were manually incorporated to constitute the dataset which was used for constructing the phylogenetic tree. These sequences were aligned using the ClustalW program [[2](#_ENREF_2)] with default parameters within the BioEdit [[3](#_ENREF_3)] software package. This alignment file was used to perform phylogenetic analysis by Maximum Likelihood method based on the Whelan And Goldman mode [[4](#_ENREF_4)] with 100 bootstrap replicates. A discrete Gamma distribution with invariable sites was used to model evolutionary rate differences. All positions containing gaps and missing data were used for tree construction. All evolutionary analyses were conducted in MEGA6 [[5](#_ENREF_5)]. The domain architectures and taxa colors were added to the final tree using iTOL [[6](#_ENREF_6)]. The domain boundaries were obtained by searching against the Pfam database [[7](#_ENREF_7)].

**References:**

1. Li W, Godzik A. Cd-hit: a fast program for clustering and comparing large sets of protein or nucleotide sequences. Bioinformatics. 2006;22(13):1658-9. doi:10.1093/bioinformatics/btl158.

2. Larkin MA, Blackshields G, Brown NP, Chenna R, McGettigan PA, McWilliam H et al. Clustal W and Clustal X version 2.0. Bioinformatics. 2007;23(21):2947-8. doi:10.1093/bioinformatics/btm404.

3. Hall TA, editor. BioEdit: a user-friendly biological sequence alignment editor and analysis program for Windows 95/98/NT. Nucleic acids symposium series; 1999.

4. Whelan S, Goldman N. A general empirical model of protein evolution derived from multiple protein families using a maximum-likelihood approach. Molecular biology and evolution. 2001;18(5):691-9.

5. Tamura K, Stecher G, Peterson D, Filipski A, Kumar S. MEGA6: Molecular Evolutionary Genetics Analysis version 6.0. Molecular biology and evolution. 2013;30(12):2725-9. doi:10.1093/molbev/mst197.

6. Letunic I, Bork P. Interactive Tree Of Life v2: online annotation and display of phylogenetic trees made easy. Nucleic acids research. 2011;39(Web Server issue):W475-8. doi:10.1093/nar/gkr201.

7. Finn RD, Bateman A, Clements J, Coggill P, Eberhardt RY, Eddy SR et al. Pfam: the protein families database. Nucleic acids research. 2014;42(Database issue):D222-D30. doi:10.1093/nar/gkt1223.
