# Supplementary figures and images for: The UBR-box and its relationship to binuclear RING-like treble clef zinc fingers
Source: Biol Direct. 2015 Jul 17;10:36. doi: 10.1186/s13062-015-0066-5 (PMC4506424; doi:10.1186/s13062-015-0066-5)

## Protein domains

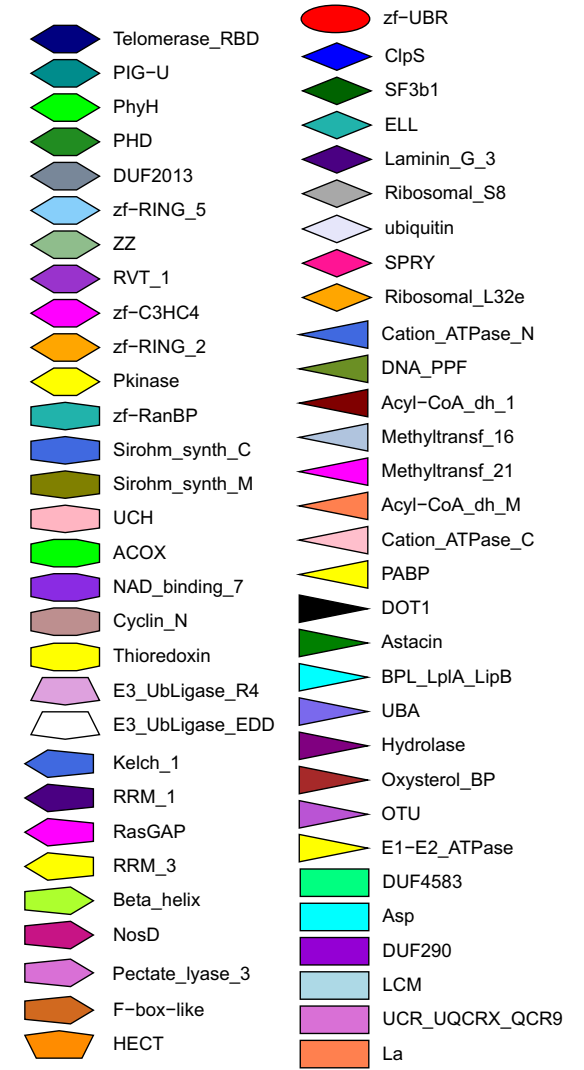

## Taxa colors

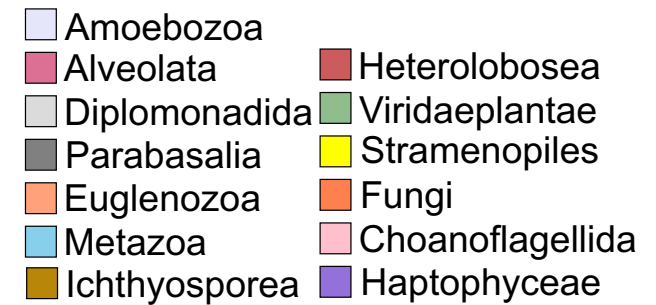

Supplement: Additional file 2: Figure S1. — Phylogenetic relationships between UBR-box domains. The figure depicts the bootstrap consensus tree for UBR-box domains from various eukaryotic taxa. Branch labels denote the UniProt ID/gi and organism name separated by ‘|’. The coloring scheme used to highlight proteins from different taxa is indicated. The domain architectures for the proteins are shown besides the branch labels. Pfam domains are represented by different shapes. [file 13062_2015_66_MOESM2_ESM.pdf]

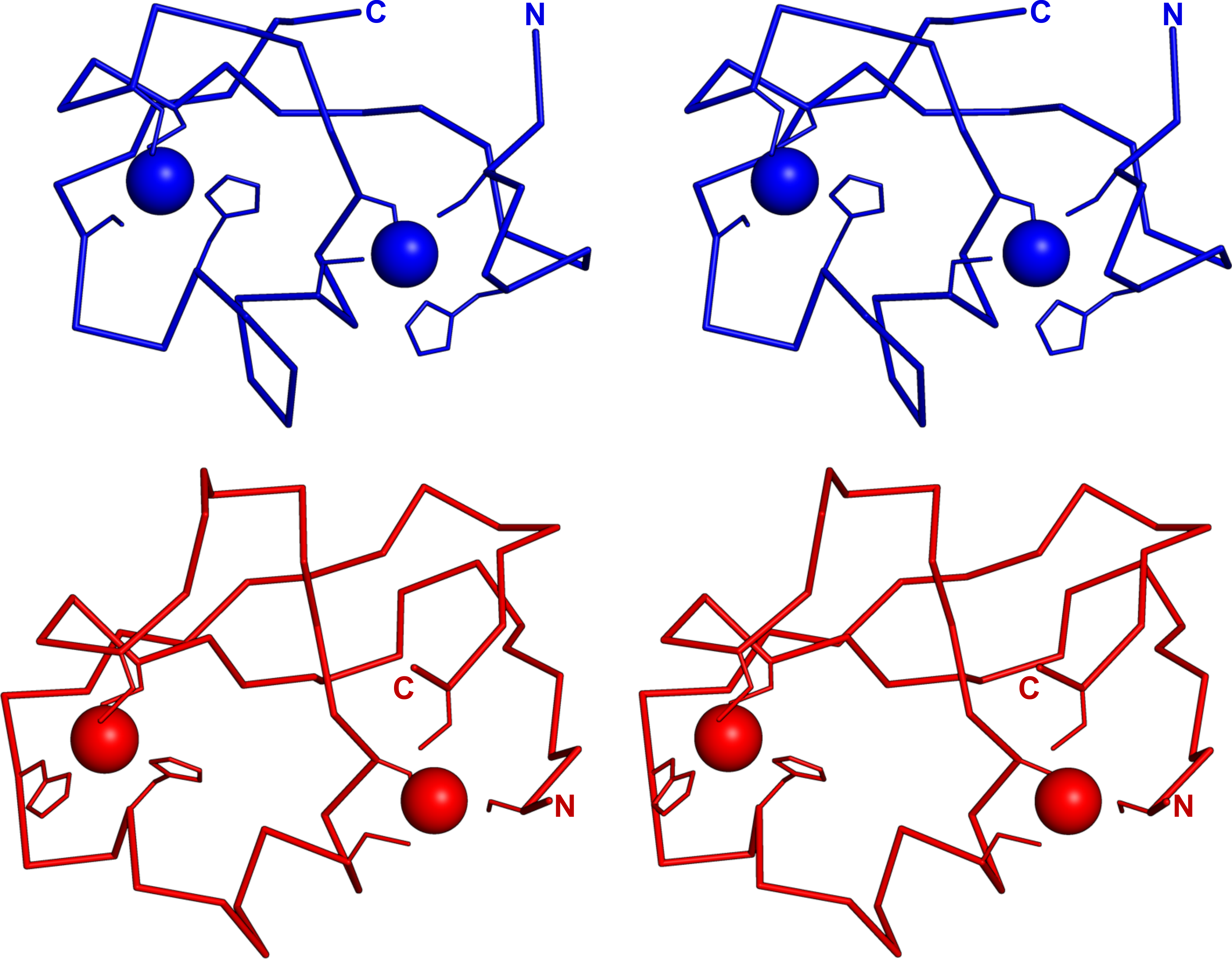

Supplement: Additional file 3: Figure S2. — Stereo diagram of the binuclear RING-like region of UBR-box and B-box. The stereo structures of the binuclear RING-like region of UBR-box from human UBR1 (PDB identifier 3NY1_A, colored red) and the B-box from E3 ubiquitin-protein ligase TRIM63 (PDB identifier 3DDT_A, colored blue) are shown. The structures were manually superimposed in PyMOL using the pair fitting command and translated thereafter. The backbone Cα chain trace is shown for both domains. The zinc ions are shown as spheres and zinc-chelating residues are shown as lines. [file 13062_2015_66_MOESM3_ESM.tiff]

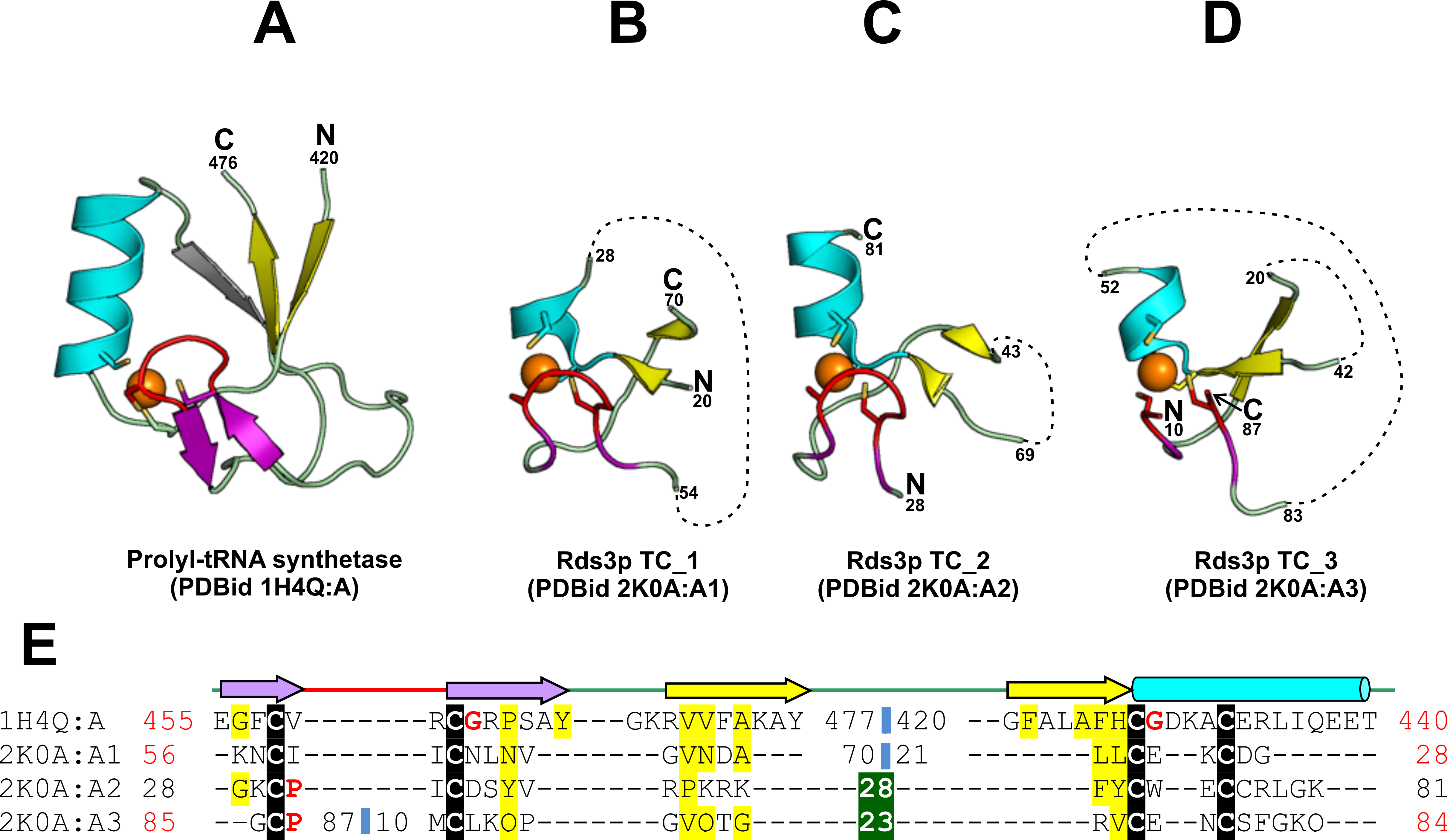

Supplement: Additional file 4: Figure S3. — Circularly permuted treble clef zinc finger domains. (A) Prolyl-tRNA synthetase C-terminal domain (PDB identifier 1H4Q_A) (B-D) Three treble clef motifs (TC_1, TC_2, TC_3) of Rds3p protein (PDB identifier 2K0A_A). TC_1 (B) and TC_3 (D) of Rds3p are permuted with respect to the classical treble clef seen in TC_2 (C) of Rds3p. The permutation seen in TC_3 is at the same position as that of the UBR-box. Coloring scheme for (A-D) follows Figure 2. (E) Structure-based multiple sequence alignment of the treble clef domains in (A-D). Coloring scheme for MSA follows Figure 3. [file 13062_2015_66_MOESM4_ESM.tiff]

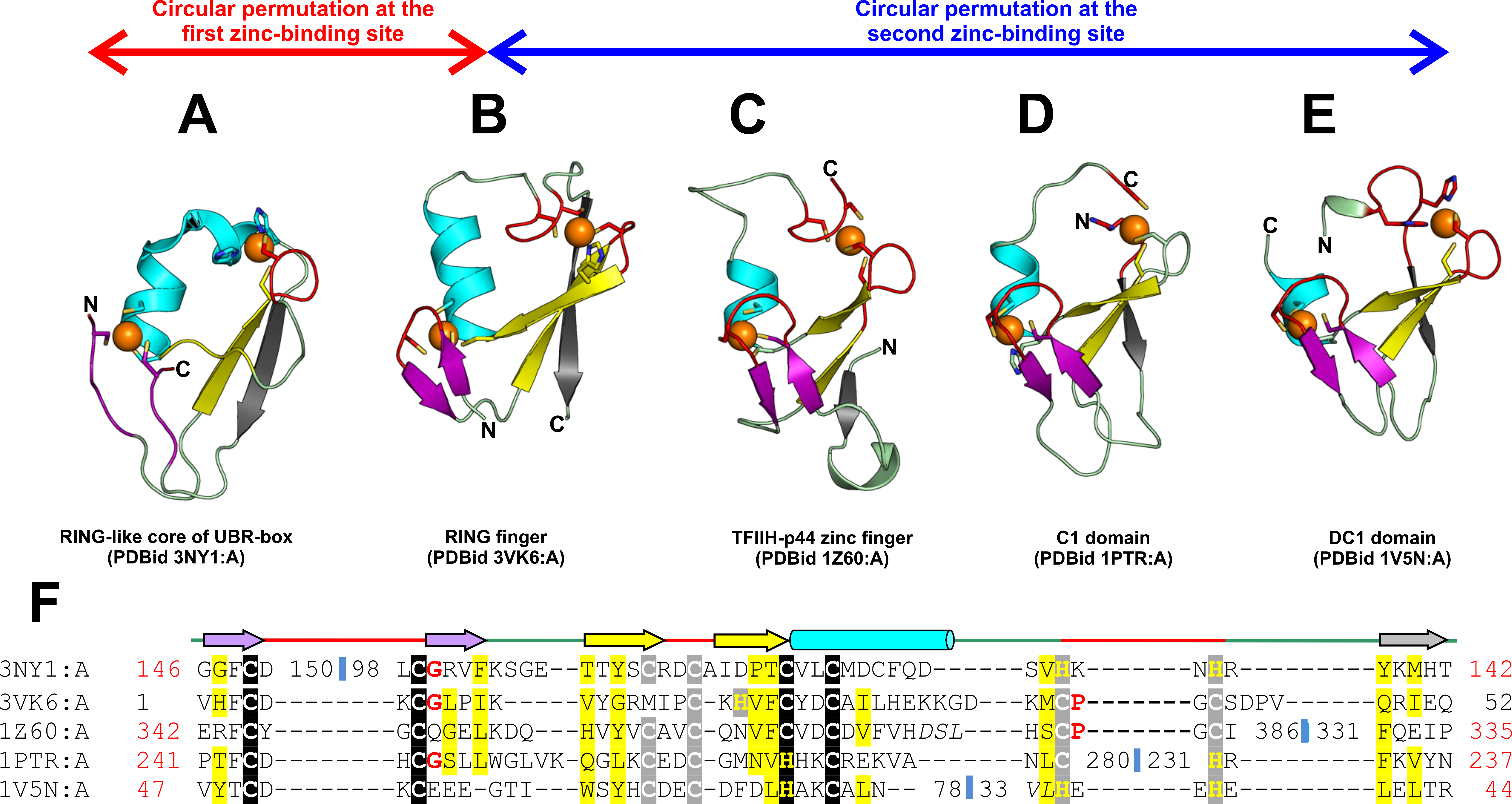

Supplement: Additional file 5: Figure S4. — Circular permutations in binuclear RING-like treble clef domains. (A) Binuclear RING-like region of the UBR-box (PDB identifier 3NY1_A) (B) RING finger domain of E3 ubiquitin-protein ligase Hakai (PDB identifier 3VK6_A) (C) Zinc finger domain of TFIIH-p44 (PDB identifier 1Z60_A) (D) C1 domain of Protein kinase C (PDB identifier 1PTR_A) (E) DC1 domain of PDI-like hypothetical protein At1g60420 (PDB identifier 1V5N_A). Coloring scheme for (A-E) follows Figure 2. (F) Structure-based multiple sequence alignment of the binuclear RING-like treble clef domains in (A-E). Coloring scheme for MSA follows Figure 3. [file 13062_2015_66_MOESM5_ESM.tiff]

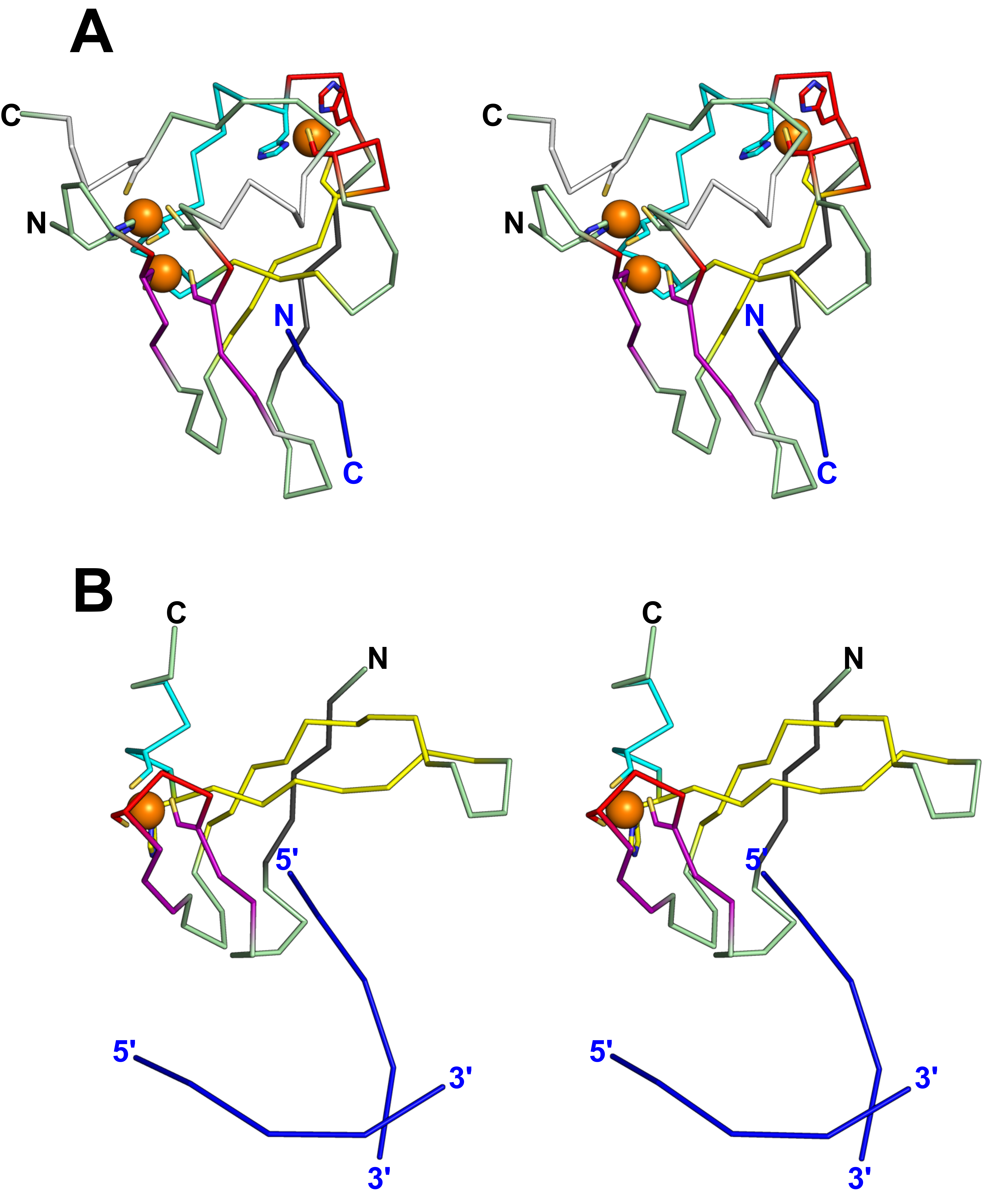

Supplement: Additional file 6: Figure S5. — Stereo diagram of ligand binding sites in UBR-box and PARP1. (A) The N-degron bound UBR-box from yeast UBR1 (PDB identifier 3NIN) (B) The dsDNA bound treble clef of PARP1 (PDB identifier 4AV1). The secondary structure elements of the treble clef domains are colored identically. Coloring scheme follows Figure 2. The bound peptide/DNA is colored dark blue. [file 13062_2015_66_MOESM6_ESM.tiff]
